# Position paper of the Cardiovascular Committee of the European Association of Nuclear Medicine (EANM) on PET imaging of atherosclerosis

**DOI:** 10.1007/s00259-015-3259-3

**Published:** 2015-12-17

**Authors:** Jan Bucerius, Fabien Hyafil, Hein J. Verberne, Riemer H. J. A. Slart, Oliver Lindner, Roberto Sciagra, Denis Agostini, Christopher Übleis, Alessia Gimelli, Marcus Hacker, on behalf of the Cardiovascular Committee of the European Association of Nuclear Medicine (EANM)

**Affiliations:** Department of Nuclear Medicine, Maastricht University Medical Center, Maastricht, The Netherlands; Cardiovascular Research Institute Maastricht (CARIM), Maastricht University Medical Center, Maastricht, The Netherlands; Department of Nuclear Medicine, University Hospital RWTH Aachen, RWTH Aachen, Aachen, Germany; Department of Nuclear Medicine, Bichat University Hospital, Inserm 1148, DHU FIRE, Assistance Publique - Hôpitaux de Paris, Paris, France; Department of Nuclear Medicine, Klinikum rechts der Isar, Technische Universität München, Munich, Germany; Department of Nuclear Medicine, Academic Medical Center, University of Amsterdam, Amsterdam, The Netherlands; Department of Nuclear Medicine and Molecular Imaging, University Medical Center Groningen, University of Groningen, Groningen, The Netherlands; Department of Biomedical Photonic Imaging, Faculty of Science and Technology, University of Twente, Enschede, The Netherlands; Nuclear Medicine and Molecular Imaging, Institute of Radiology, Heart and Diabetes Center NRW, Bad Oeynhausen, Germany; Nuclear Medicine Unit, Department of Experimental and Clinical Biomedical Sciences, University of Florence, Florence, Italy; Department of Nuclear Medicine, CHU Côte de Nacre, Normandie Université, Caen, France; Department of Clinical Radiology, Ludwig-Maximilians Universität München, München, Germany; Fondazione Toscana Gabriele Monasterio, Pisa, Italy; Division of Nuclear Medicine, Department of Biomedical Imaging and Image-guided therapy, Medical University Vienna, Vienna, Austria; Department of Nuclear Medicine and Cardiovascular Research Institute (CARIM), Maastricht University Medical Center (MUMC+), P. Debyelaan 25, 6229 HX Maastricht, The Netherlands

**Keywords:** Atherosclerosis, Positron emission tomography, Position paper

## Abstract

Cardiovascular diseases are the leading cause of death not only in Europe but also in the rest of the World. Preventive measures, however, often fail and cardiovascular disease may manifest as an acute coronary syndrome, stroke or even sudden death after years of silent progression. Thus, there is a considerable need for innovative diagnostic and therapeutic approaches to improve the quality of care and limit the burden of cardiovascular diseases. During the past 10 years, several retrospective and prospective clinical studies have been published using ^18^F-fluorodeoxyglucose (FDG) positron emission tomography (PET) to quantify inflammation in atherosclerotic plaques. However, the current variety of imaging protocols used for vascular (arterial) imaging with FDG PET considerably limits the ability to compare results between studies and to build large multicentre imaging registries. Based on the existing literature and the experience of the Members of the European Association of Nuclear Medicine (EANM) Cardiovascular Committee, the objective of this position paper was to propose optimized and standardized protocols for imaging and interpretation of PET scans in atherosclerosis. These recommendations do not, however, replace the individual responsibility of healthcare professionals to make appropriate decisions in the circumstances of the individual study protocols used and the individual patient, in consultation with the patient and, where appropriate and necessary, the patient’s guardian or carer. These recommendations suffer from the absence of conclusive evidence on many of the recommendations. Therefore, they are not intended and should not be used as "strict guidelines" but should, as already mentioned, provide a basis for standardized clinical atherosclerosis PET imaging protocols, which are subject to further and continuing evaluation and improvement. However, this EANM position paper might indeed be a first step towards "official" guidelines on atherosclerosis imaging with PET.

## Background

Cardiovascular diseases (CVD) are the leading cause of death in Europe (5 million deaths per year) and in the world (19 million deaths per year) irrespective of low- or middle-income countries [1, 2]. More than one in three adults in Western countries die from coronary artery disease and about 25 % from stroke. The cost of CVD in the European Union was €196 billion in 2009. There has been considerable progress in prevention and treatment of CVD. Prevention of CVD has largely been achieved through treatment of risk factors (in particular treatment of dyslipidaemia with statins), and development of potent anticoagulant and antiplatelet therapies. Improved treatment of CVD has been possible through the widespread availability of balloon angioplasty and stent implantation as well as cardiac surgical procedures for the local treatment of occluded or highly stenotic arteries. Preventive measures, however, often fail and CVD may manifest as an acute coronary syndrome, stroke or even sudden death after years of silent progression [3].

Thus, there is a considerable need for innovative diagnostic and therapeutic approaches to improve the quality of care and limit the burden of CVD.

Atherosclerosis is responsible for the vast majority of heart attacks, strokes and peripheral vascular disease. It is characterized by accumulation of lipids, inflammatory cells and connective tissue within the arterial wall leading to the formation of atherosclerotic plaques [4–6]. Atherosclerotic changes in the vessel wall reflect a chronic, progressive disease, which has a long asymptomatic phase, and atherosclerotic plaques can remain quiescent for years but can become life threatening when they rupture. Rupture of such a plaque (a so-called vulnerable plaque) initiates clot formation in the vessel lumen which disturbs blood flow and from which emboli may break off and lodge in the downstream circulation. Plaque rupture can lead ultimately to acute myocardial infarction or stroke. Unfortunately, no evaluated biomarker or imaging technique is available that can predict the risk in a particular patient of plaque rupture and a subsequent acute cardiovascular event. Whether and how a plaque ruptures is determined by its macroscopic structure and its microscopic composition [7]. Arterial wall inflammation seems to play a key role in atherosclerotic plaque rupture leading to clinical cardiovascular events. Therefore, detection of arterial inflammation with imaging is an attractive approach to identifying patients at highest risk of plaque rupture [8].

^18^F-fluorodeoxyglucose (FDG) is a glucose analogue that is taken up by cells with high metabolic activity such as inflammatory and tumour cells. Its uptake in tissues can be quantified by positron emission tomography (PET) imaging. Accumulation of FDG in the arterial wall is thought to reflect increased inflammation in atherosclerotic plaques [9, 10]. During the past 10 years, several retrospective and prospective clinical studies have used FDG PET imaging for the quantification of the intensity of inflammation in atherosclerotic plaques. Signal quantification in the vessel wall measured with FDG PET is, however, strongly influenced by the parameters selected for PET image acquisition including delay between FDG injection and imaging, the approach used for signal quantification and the use of correction factors. The current variety of imaging protocols used for vascular (arterial) imaging with FDG PET considerably limits the ability to compare results between studies and to build large multicentre imaging registries.

The objective of this position paper was to review the different parameters that might influence FDG uptake in atherosclerotic plaques, and propose optimized standardized scan protocols for imaging atherosclerosis with PET based on the existing literature and the experience of the members of the EANM Cardiovascular Committee. We limited this position paper to the imaging of atherosclerosis in relatively larger arterial vessels. Imaging of the coronary arteries is only briefly mentioned and is not discussed in full here. With this position paper, we hope to standardize the practice of arterial PET imaging in European nuclear medicine centres and thereby facilitate (multicentre) clinical studies on the imaging of atherosclerosis with PET. In addition this harmonization of protocols would ultimately allow better extrapolation of acquired data and therefore optimized applicability of this imaging modality in the setting of atherosclerosis.

## Technical recommendations for imaging atherosclerosis with FDG PET

### FDG: dose, timing after injection (circulation time), prescan fasting glucose

#### Injected dose of FDG

The dose of FDG administered in clinical studies varies considerably ranging from 185 MBq to 925 MBq [10–12]. Factors to take into consideration to define the dose of FDG injected are the sensitivity of the PET scanner (in particular, 2D vs. 3D acquisitions), the duration of imaging steps, the body weight of the patient, and the delay between tracer injection and image acquisition. As for other clinical indications, the ALARA (As Low As Reasonably Achievable) principle should apply to vascular imaging studies. The exposure of patients to radiation should be kept as low as possible, in particular in studies monitoring changes of FDG uptake in atherosclerotic plaques over time or the effects of treatments requiring repeated FDG PET imaging studies. Only one study has directly evaluated the impact of the injected FDG dose in vascular FDG PET imaging in atherosclerosis [13]. In this study, the injection of a lower dose of FDG had neither an impact on the quantification of FDG uptake (standardized uptake value, SUV, and target to background ratio, TBR), nor an impact on the classification of patients into tertiles based on the intensity of FDG uptake [13].

*Section summary and recommendations:*

- Considering a circulation time of 2 h for vascular imaging (see below), we recommend that the injected activity of FDG should be between 3 and 4 MBq/kg body weight for atherosclerotic plaque imaging to allow sufficient image quality. In patient screening and/or repeated PET studies, lower FDG activities need to be considered to limit the patient's total radiation exposure. Further studies are required to evaluate whether lower FDG activities leading to lower exposure of patients to radiation still provide appropriate image quality.

#### Delay between FDG injection and PET acquisitions (FDG circulation time)

For arterial imaging with PET, longer delays between FDG injection and PET imaging than those used for oncology are recommended to allow sufficient FDG accumulation in the arterial wall and to reduce the intensity of FDG signal in the blood. The FDG circulation time is critical as optimal contrast between the target (plaque, arterial wall) and the background (blood) is essential to ensure accurate quantification of plaque FDG uptake [9, 11, 13–19]. Improved target-to-background contrast is typically achieved by delayed imaging [13, 20]. In addition, vascular imaging with PET is subject to partial volume effects (PVE). The intensity of the signal measured in the arterial wall with FDG PET imaging is strongly influenced by the adjacent background signal from circulating blood. Therefore, the blood signal should be kept as low as possible for precise quantification of FDG uptake in atherosclerotic plaques.

Dynamic PET studies have been performed both in patients with carotid artery disease and in animal models of atherosclerosis [9, 11, 13, 14, 21]. At earlier time-points (1 h), the contrast between FDG uptake in the vessel wall and the residual blood signal is suboptimal leading to underestimation of FDG uptake in the vessel wall, whereas at later time-points (2.5 – 3 h) FDG uptake measured with PET approaches the true vascular FDG uptake [9]. Delays between FDG injection and imaging of >3 h do not provide any additional improvement in the quantification of FDG uptake in the vessel wall. Tawakol et al. using a delay between FDG injection and PET acquisition of 3 h found excellent correlations between the intensity of FDG uptake measured in carotid atherosclerotic plaques and the density of macrophages measured on corresponding histological sections obtained after carotid endarterectomy [9].

*Section summary and recommendations:*

- Based on these results, we recommend the acquisition of PET images 2 h after injection for reliable quantification of FDG uptake in the arterial vessel wall and/or plaques. In our opinion, this imaging delay represents the best compromise between a low background signal in blood and an acceptable duration of the PET study for patients.

#### Prescan fasting glucose

In oncological studies, the intensity of FDG uptake in tumour tissues is influenced by the patient's prescan glucose levels [22–24]. The observed reduction in FDG uptake is most likely due to competition between glucose and FDG as facilitative transport via the glucose transporter protein (GLUT) system is the most important way by which both glucose and FDG enter human cells [25]. Additionally, the intensity of FDG uptake in macrophages in cell culture depends not only on the duration of culture and the degree of macrophage activation but also on the prevailing glucose concentration, such that FDG uptake decreases with increasing glucose concentration in the culture medium [25]. As diabetes is a major risk factor for atherosclerotic disease, the problem of elevated prescan glucose levels occurs frequently in patients scheduled for FDG PET imaging of vessel wall inflammation.

The impact of glucose levels on FDG uptake in inflammatory lesions has been less studied than in tumour cells. Cell culture experiments suggest that moderate hyperglycaemia (up to 250 mg/dl, 14 mmol/l) does not adversely affect FDG uptake in inflammatory cells [23, 26]. In contrast, data from experimental studies in rats show a significant reduction in FDG uptake in the lesions of infectious and noninfectious inflammatory models with moderate hyperglycaemia (150 – 180 mg/dl, 8.3 – 10.0 mmol/l) but also, after glucose loading, decreased GLUT-1 and GLUT-3 expression in infectious lesions [27, 28].

In a prospective study by Bucerius et al. [13], a negative correlation was found between prescan glucose levels and SUV and TBR values measured in the ascending aorta and the carotid arteries. Elevated prescan glucose values were associated with increased FDG blood pool activity [13]. As blood pool activity is used as the denominator in calculating TBR, this might explain at least in part the lower TBR values measured in patients with high prescan glucose values. Furthermore, prescan glucose levels higher than 126 mg/dl (7.0 mmol/l) have been found to be associated with lower TBR values, whereas prescan glucose values below this level did not show any effect on TBR values [13].

In a patient with a blood glucose level above 126 mg/dl (7.0 mmol/l) approaches to lower the patient's glucose level could be considered [29]. Such approaches include administration of rapid-acting insulin with an appropriate time of 4 h between administration of insulin and injection of FDG or asking the patient to hydrate while ambulating and recheck the blood glucose level periodically until an acceptable level has been achieved. However, all of these approaches relate to oncological FDG PET imaging and are included in the EANM recommendations for oncological PET imaging, and data regarding their performance in atherosclerosis imaging with FDG PET are still lacking [29]. This also holds true for the preparation of patients with type II diabetes mellitus in whom an appropriate preparation before the scan is even more crucial, particularly if intravenous administration of contrast agent is necessary. In these circumstances, if the patient is taking metformin, this medication should be discontinued at the time of the procedure and withheld for 48 h after the procedure under the appropriate control of the referring physician [29]. As mentioned above, due to a lack of data regarding the optimal protocols for reducing high glucose levels in patients scheduled for atherosclerotic FDG PET imaging, we refer to the EANM recommendations for oncological patients [29]. However, until sufficient data are available to provide specific recommendations for glucose adjustment in vascular patients, these recommendations should be applied somewhat cautiously in patients undergoing atherosclerotic FDG PET imaging.

In patients in whom blood glucose levels below approximately 130 mg/dl (approximately 7.0 – 7.2 mmol/l) cannot be achieved using the approaches mentioned above, a correction of the vascular FDG uptake according to the EANM recommendations for oncological PET imaging can be considered [29]. This approach was applied on a previous study on atherosclerotic PET imaging using a dedicated formula normalizing the measured glucose content for an overall population average of 90 mg/dl (5.0 mmol/l): SUV_gluc_ = SUV × patient's blood glucose in milligrams per decilitre (mmol/l)/90 mg/dl (5.0 mmol/l) [30].

*Section summary and recommendations:*

- Taken together, these studies suggest that arterial imaging should ideally be performed in patients with prescan glucose levels lower than approximately 130 mg/dl (approximately 7.0 – 7.2 mmol/l). In patients in whom these blood glucose levels cannot be achieved by other approaches as described above, correction of the vascular FDG uptake according to the EANM recommendations for oncological PET imaging can be considered. However, this needs to be validated more extensively in the setting of vascular wall imaging with FDG PET.

### PET reconstruction protocols for vascular imaging in atherosclerosis

Accurate estimation of radiotracer uptake in arterial lesions is extremely challenging given the small size of the lesions compared to the spatial resolution of PET. In particular, uptake measurements in the arterial walls are strongly affected by PVE, which cause large activity underestimation in structures that are typically less than three times the spatial resolution in the reconstructed images [31]. Assuming a constant uptake in a lesion, the bias in uptake measurements introduced by PVE depends on a number of factors, including the volume of the lesion, its shape and contrast with respect to surrounding tissue, the spatial resolution in the PET images and motion artefacts. It also depends on how the uptake is measured [32, 33]. The smaller the lesion, the greater the underestimation of activity in that lesion, since activity within the lesion spills out of the lesion due to the blurring caused by the point spread function (PSF) in the reconstruction algorithm of the PET imaging system. In addition, when lesion activity is very different from that of surrounding tissues (high contrast), the spill out is not compensated for by the spill-in of activity located outside the lesion. Conversely, when the lesion is large, PVE is not so severe [31]. Using simulated lesions, Huet et al. found that the true SUV in atherosclerotic plaques is largely underestimated, up to a factor of 7 whatever the PET acquisition and reconstruction protocol [34]. This is particularly true when compared to the spatial resolution of the reconstructed images (around 5 mm), since the FDG signal is located in a relatively small area (<1 mm) within the atherosclerotic lesion.

Despite the strong underestimation of the FDG signal in atherosclerotic lesions, several PET acquisition parameters can be optimized for arterial imaging to limit this bias. Overall, for a standard deviation in the measurement of less than 0.5 SUV units, the lowest bias was always found using an 8-min PET acquisition and reconstruction including a PSF model using a voxel size of 1 × 1 × 1 mm and no postfiltering, with at least 120 iterations when using an OSEM approach (Table 1). If these acquisition parameters are used the deviation in SUV is less dependent on the dimension of the atherosclerotic lesion [33, 34].

**Table 1:** PET imaging parameters in atherosclerosis

Study design	Type of study	FDG dose (MBq/kg body weight)	Circulation time (h)	Prescan glucose value (mmol/l)	Reconstruction protocol	Regions to be analysed	FDG uptake parameters^d^
Prospective	General^a^	3 – 4	2	≤7.0	Acquisition setup 8 min, reconstruction with PSF, 1 × 1 × 1 mm voxel size, no postfiltering, at least 120 iterations using OSEM	CC, AA, DA, AArch, AbdA, Iliac, Fem	Mean TBR_mean_, mean TBR_max_
	Identifying patients at high risk of a cardiovascular event from oncological studies					CC, AA, AArch^c^	Mean TBR_mean_, mean TBR_max_
	Using arterial inflammation as a surrogate endpoint for interventional studies					CC, AA, DA, AArch, AbdA, Iliac, Fem^c^	Mean TBR_max_, MDS, AS
	Identifying high-risk carotid lesions					CC	MDS, AS
Retrospective	Identifying patients at high risk of a cardiovascular event from oncological studies	≥2	≥1	≤7.0^b^	Best retrospectively applicable reconstruction protocol	CC, AA, AArch^c^	Mean TBR_mean_, mean TBR_max_

*TBR* target to background ratio, *MDS* most diseased segment, *AS* active segments, *CC* common carotid artery, *AA* ascending aorta, *DA* descending aorta, *AArch* aortic arch, *AbdA* abdominal aorta, *Iliac* iliac artery, *Fem* femoral artery

^a^More generalized inflammatory changes in the context of atherosclerosis; for example, studies assessing correlations between arterial FDG uptake and clinical risk factors, and studies evaluating the systemic inflammatory burden in patients with arterial atherosclerotic inflammation

^b^Patients with prescan glucose values >126 mg/dl (7 mmol/l) should be excluded from further analysis, or correction of the SUV based on dedicated formulas should be considered

^c^Threshold TBR value for identification of patients with increased arterial inflammation and/or cardiovascular risk >1.6 (see also section Parameters for quantification of radiotracer uptake in atherosclerotic plaques)

^d^TBR carotid artery: *artery* mean SUV_mean_, mean SUV_max_; *blood pool* mean SUV_mean_ jugular vein

TBR ascending aorta: *artery* mean SUV_mean_, mean SUV_max_; *blood pool* mean SUV_mean_ superior vena cava

TBR aortic arch: *artery* mean SUV_mean_, mean SUV_max_; *blood pool* mean SUV_mean_ superior vena cava

TBR descending aorta: *artery * mean SUV_mean_, mean SUV_max_; *blood pool* mean SUV_mean_ inferior vena cava

TBR iIiac artery: *artery* mean SUV_mean_, mean SUV_max_; *blood pool* mean SUV_mean_ inferior vena cava

TBR femoral artery: *artery* mean SUV_mean_, mean SUV_max_; *blood pool* mean SUV_mean_ inferior vena cava

However, even with these optimized parameters, the mean error in SUV remained high because of PVE. Several strategies might be considered in the future to reduce these errors: improving the spatial resolution of the PET images based on hardware development and implementing explicit partial volume correction and motion correction, especially in coronary arteries [33, 35].

*Section summary and recommendations:*

- Reconstruction parameters for PET images may influence SUV and TBR quantification. We therefore recommend the implementation of dedicated PET acquisition, processing and reconstruction protocols for arterial imaging as described above and in Table 1*.*

### Parameters for quantification of radiotracer uptake in atherosclerotic plaques

Accurate quantification of radiotracer activity in arterial wall or plaque on PET images can be problematic. SUV (i.e. the decay-corrected tissue concentration in in kilobecquerels per millilitre divided by the injected dose per body weight in kilobecquerels per gram) is the most commonly used parameter for measuring lesion activity in patients undergoing PET/CT imaging for the initial diagnosis, staging or evaluation of treatment response of malignancies. Maximal SUV (SUV_max_) is the most intense voxel activity within a region of interest (ROI) while mean SUV (SUV_mean_) is the mean SUV within the ROI. On the one hand, SUV_mean_ is not a realistic measure in atherosclerotic plaque quantification because it is almost impossible to accurately define the edge of an arterial plaque on CT images. On the other hand, SUV_max_ may not accurately represent the lesion’s overall activity as tracer distribution is often not homogeneous within a lesion. SUV_max_ is, however, the most commonly used parameter to quantify FDG uptake in atherosclerotic plaques. SUV_max_ measurements in plaques reflect the part of the lesion with the highest FDG uptake. The level of noise in the image and the adjacent blood signal (spill-in of adjacent activity related to PVE) can influence measurements of SUV_max_.

 As discussed above, quantification of FDG uptake in vascular imaging using SUV_max_ is improved by the use of late time-points and long acquisition times. SUV_max_ values measured in atherosclerotic plaques tend to underestimate the true FDG uptake by a factor up to 7 depending on the size of the lesion as compared to the spatial resolution of the system. In addition, overcorrection of heavily calcified arteries by low-dose CT might also lead to an underestimation of the true FDG uptake. Development of algorithms for correction of PVE in arterial lesions in association with improved identification of plaque contours based on high-resolution, high-contrast MR images, which are now becoming available with combined PET/MRI systems, might help to quantify more precisely the intensity of radiotracer uptake in atherosclerotic plaques [36].

An unique parameter that is used only in vascular imaging is the TBR, which was used for the first time in arterial FDG PET imaging in 2006, and which has since been widely used in several studies [9]. The TBR_max_ is calculated as the ratio of SUV_max_ measured in atherosclerotic plaques or the arterial vessel wall and venous blood pool mean SUV_mean_ to correct for blood-pool uptake. TBR measured in atherosclerotic plaques on FDG PET/CT has been shown to correlate well with plaque macrophage density on corresponding histological sections [9, 10, 37–39]. In addition, TBR values measured in different arterial territories demonstrate excellent intrareader and interreader agreement in patients imaged twice with FDG PET with an interval of 2 weeks between studies [40]. TBR is robust with different scan settings (acquisition times and reconstruction filters) and tracer circulation times, while SUV might show significant differences with each of these settings [38, 41].

 TBR_mean_ and TBR_max_ have both been shown to be highly reproducible [42]. However, reproducibility of a measurement does not equal accuracy. On the one hand, TBR is calculated as a ratio of two SUVs. TBR therefore compensates for errors in patient weight, the dose of radiotracer injected and imaging time-point. On the other hand, TBR varies as a function of blood-pool activity (denominator). Blood-pool activity can be affected by many factors including: (1) FDG uptake in circulating blood cells, (2) chronic renal insufficiency, and (3) blood glucose levels [38, 43]. In addition, measurement of blood SUV_mean_ can sometimes be challenging. Care should therefore be taken to avoid any interference with high FDG uptake in tissues adjacent to the right atrium (myocardium), to the jugular vein (muscle, lymph node) and to the superior vena cava (myocardium, aorta) when measuring blood SUV_mean_. The SUV_mean_ should be measured by drawing small ROIs in venous structures (jugular veins, superior or inferior vena cava) in different locations within the venous target vessel and then averaged to approach one single most accurate value for circulating blood-pool signal [38]. It has to be pointed out that the FDG blood-pool activity should be measured in a venous structure close to the target artery allowing data acquisition in the same bed position of the PET scan. This ensures that the delay between injection of tracer and data acquisition, and consequently tracer uptake in the arterial target structure and the washout of tracer from the venous blood pool, will be the same for the arterial and the venous vessel. Otherwise a bias in the calculation of the TBR cannot be excluded.

The use of other structures or organs for measurement of background activity, such as the liver, muscles, spleen and adipose tissue, should be considered cautiously because of the systemic character of atherosclerosis, which might lead to altered FDG accumulation in these structures and, consequently, to altered final results [38, 44–49].

It has been shown that arterial FDG uptake significantly varies among arterial territories [40, 42]. In general, the mean TBR of the carotid arteries is significantly higher than in all aortic territories (ascending and descending aorta, aortic arch and abdominal aorta) and the iliac and femoral arteries [40, 42]. In contrast, no significant difference has been observed between the FDG uptake in the left and right carotid artery [42]. These differences clearly have to be kept in mind, as when comparing different scans intraindividually or interindividually, the arterial FDG uptake needs to be compared between the same arterial territories to avoid bias related to the intrinsically different arterial FDG uptake patterns. This holds true for both a "per-region/per-territory" as well as a "per-patient" analysis. Mainly for the latter, it is important not to compare the highest FDG uptake identified in one patient scan with the highest in another scan of the same or another patient independent of the arterial territory (for example, carotid artery versus aortic arch, etc.) because of the intrinsic differences in FDG uptake between different arterial regions.

**Fig. 1:**
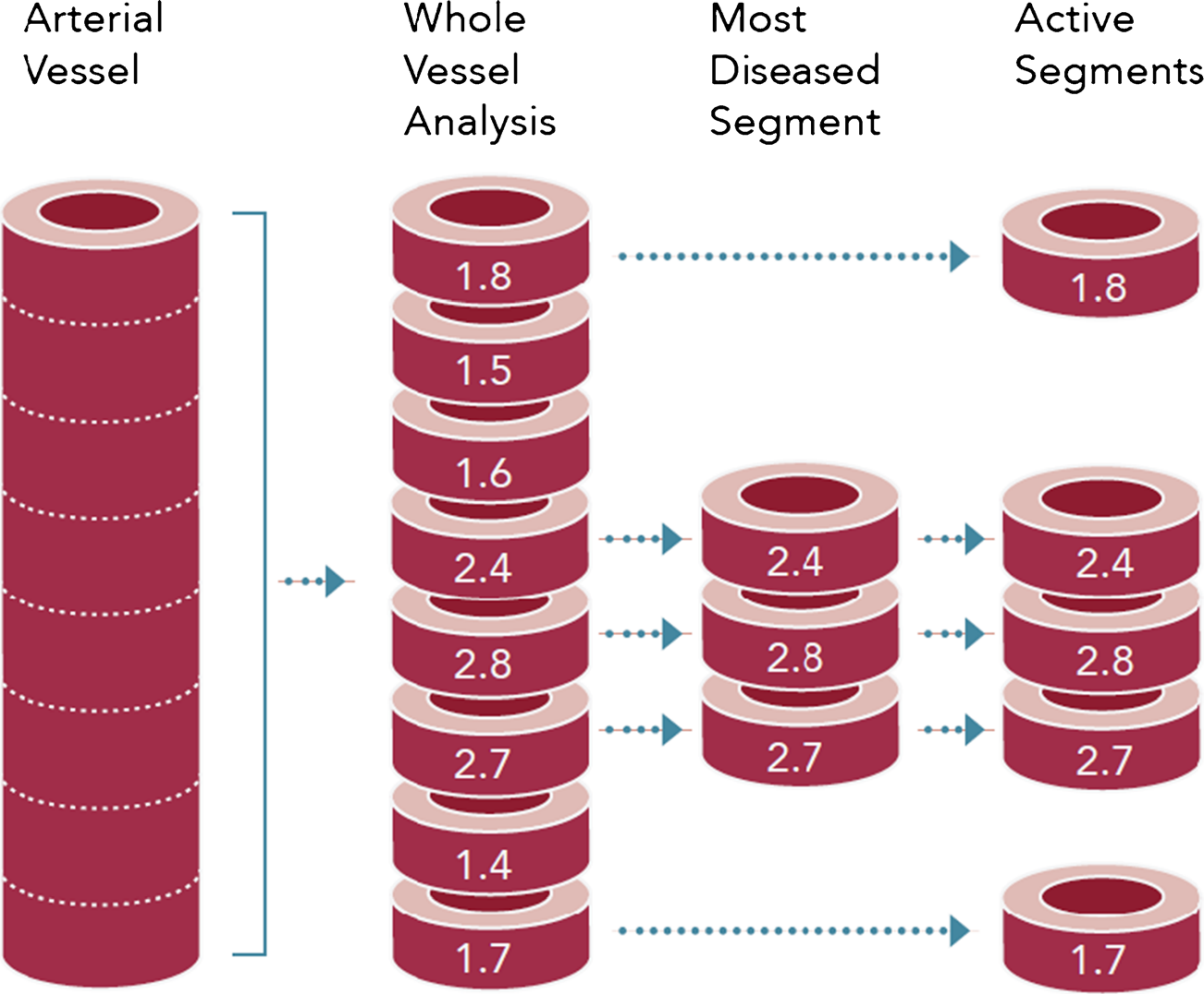
Current most frequently used approaches to quantifying arterial FDG uptake in clinical studies. All values given are maximal target to background ratios (TBR_max_). A TBR threshold of >1.6 was considered significant for the active segment analysis. The mean TBR_max_ of the whole vessel would be 1.99, and the mean TBR_max_ of the most diseased segment would be 2.63 (based on a graph by Tawakol et al. [64]). Image courtesy © Annette Bucerius

On basis of the TBR calculation, three different approaches for the quantitative assessment of arterial FDG uptake in a target (index) vessel have been developed and tested in clinical trials [9, 13, 19, 30, 40, 42, 44, 47, 50]. The first approach involves measuring the average TBR (TBR_mean_ or TBR_max_, see below) along all the axial segments that comprise the target vessel (whole vessel mean TBR_mean_ or TBR_max_; Fig. 1) [9, 13, 30, 40, 42, 44, 50]. This approach might be intrinsically more robust and less sensitive to image noise and to FDG signal originating from structures adjacent to the vessel wall as the signal will be averaged over a large number of segments. On the other hand, this approach will be less sensitive for detecting small changes over time in FDG uptake in focal areas with atherosclerotic plaques [42, 51]. However, the approach is well suited to the assessment of global vascular inflammation in patients as a marker of cardiovascular risk and for comparison with other surrogate markers of CVD (Table 1).

The second approach involves measuring the average TBR_max_ in a small segment of the index vessel with the highest FDG uptake, the most diseased segment (MDS; Fig. 1). In the study by Tawakol et al. [50], the MDS was defined as a 1.5-cm arterial segment centred on the slice of the target artery demonstrating the highest FDG uptake (as quantified by TBR_max_). The FDG uptake within the MDS was then calculated as a mean of the TBR_max_ values from three contiguous axial segments [50]. This approach was particularly well suited to evaluating the degree of inflammation in a particular atherosclerotic plaque in an individual patient (Table 1).

The third approach, active segment (AS) analysis, is based on the methods used by Tahara et al. [19] and Elkhawad et al. [47] and was applied by Emami et al. in a clinical trial (Fig. 1) [52]. In this approach only arterial locations with a high predefined FDG uptake at baseline are analysed. Only lesions with increased FDG uptake (in the studies by Emami et al. and Elkhawad et al. a TBR of ≥1.6) are then used to evaluate the effect of therapeutic interventions on FDG uptake [19, 47, 52]. The optimal cut-off value indicating arterial segments, plaques or arteries with actual inflammation is, however, still a matter of debate, and needs to be defined. Previous FDG PET imaging studies with pathological correlations demonstrate that a TBR value <1.6 is associated with <5 % inflammation within the atheroma [12, 53, 54]. However, it has yet to be proven whether <5 % inflammation is not associated with an increased cardiovascular risk. Irrespective of these considerations, the AS approach, which focuses only on the plaques with the most severe inflammation, might improve the sensitivity of FDG PET imaging in identifying changes in FDG uptake in atherosclerotic plaques in relation to the tested intervention (Table 1).

*Section summary and recommendations:*

- For the quantification of FDG uptake in atherosclerotic plaques, we recommend using TBR instead of SUV as the use of a ratio between two measurements limits the effects on signal quantification of errors in patient weight and in the dose of radiotracer injected and of the imaging time-point.
- When comparing arterial FDG uptake between different scans the same arterial territory should be compared because of the intrinsically variable FDG uptake patterns in different arterial regions, which may bias the results of, for example, trials investigating the effect of interventions on arterial FDG uptake.
- The choice of parameters used for quantification of FDG uptake in the vascular wall depends on the aim of the study: TBR_mean_ and TBR_max_ are well suited to the assessment of global vascular inflammation in patients as markers of cardiovascular risk and to comparison with other surrogate markers of CVD. The MDS is a relevant approach when evaluating the degree of inflammation in an individual atherosclerotic plaque in a patient. The effectiveness of therapeutic interventions on FDG uptake in plaques might be improved by focusing on the arterial segments with the highest FDG uptake at baseline using metrics such as mean TBR_max_, MDS and AS.

## Clinical relevance of FDG PET in atherosclerosis

### Prognostic value of arterial FDG uptake

The prognostic value of FDG PET imaging of atherosclerosis in predicting cardiovascular events was first tested retrospectively in cohorts of patients who were undergoing FDG PET imaging for oncological indications [41, 55–57]. Rominger et al. found that in a large series of almost 1,000 patients imaged with FDG PET for cancer, the intensity of FDG uptake in large arteries was a strong predictor of a subsequent vascular event [55]. Furthermore, patients with extensive calcification of large arteries and high FDG uptake were at highest risk of a cardiovascular event during follow-up [55]. Figueroa et al. evaluated the incremental value of vascular PET imaging for predicting cardiovascular events beyond the Framingham risk score (FRS) [41]. In 514 individuals who fulfilled the defined inclusion criteria of no prior CVD, absence of cancer and ≥30 years of age, the intensity of FDG uptake was quantified using TBR in the ascending aorta. Cardiovascular events were defined as incident stroke, transient ischaemic attack, acute coronary syndrome, revascularization, new-onset angina, peripheral arterial disease, heart failure, or cardiovascular related death. During a median follow-up period of 4.2 years, 44 participants had a cardiovascular event. The TBR not only predicted the cardiovascular events independent of traditional risk factors, but in addition to the FRS score improved the risk evaluation in this population [41]. Marnane et al. prospectively evaluated 60 patients with recent stroke, transient ischaemic attack, or retinal embolism and ipsilateral carotid stenosis (≥50 %) who had been imaged with FDG PET [56]. Patients with the highest FDG uptake in the ipsilateral carotid plaques were at the highest risk of an early recurrent stroke during follow-up. The only independent predictor of stroke recurrence using a Cox regression model in this small group of patients was high FDG uptake in carotid plaques [56].

### FDG PET as surrogate endpoint in intervention studies

#### Effects of statin therapy on FDG uptake in arterial plaques

Tahara et al. imaged 43 cancer patients with FDG PET before and after 3 months of low-dose statin (simvastatin 20 mg daily) or placebo treatment. This study was the first to show that a significant decrease in the intensity of FDG uptake can be detected as early as 3 months after the introduction of statin therapy [19]. In two other studies the effect of statins on arterial FDG uptake was investigated. In the first study a 7.9 % reduction in aortic FDG TBR was observed after 6 months of treatment with 20 mg per day of atorvastatin. In the second study, a significant reduction in FDG signal following treatment with atorvastatin was mirrored by a decrease in the levels of inflammatory biomarkers, including C-reactive protein (CRP) measured using a high-sensitivity assay (hs-CRP) [58–60]. Intensification of statin therapy with atorvastatin from 10 mg to 80 mg per day led to a rapid reduction in atherosclerotic inflammation as revealed by FDG PET 4 and 12 weeks after start with statin therapy [50]. Interestingly, changes in TBR did not correlate with lipid profile changes, indicating a lipid-independent antiinflammatory effect of statins [50]. These clinical studies with statin therapy pave the way for the use of FDG PET imaging for the evaluation of new drugs aimed at reducing inflammatory activity in the arterial wall and at plaque stabilization.

#### Evaluation of the effects of new antiatherosclerotic drugs on arterial FDG uptake

The studies discussed above have increased the interest of the pharmaceutical industry in FDG PET imaging for the evaluation of new antiatherosclerotic drugs. The dal-PLAQUE study is an interesting example of the potential role of plaque imaging in the evaluation of new drugs [61, 62]. In this study, participants were randomly assigned to receive the HDL cholesterol-raising drug dalcetrapib or placebo. FDG PET was performed at baseline and after 3 and 6 months treatment to evaluate the effects of dalcetrapib on FDG uptake in aortic and carotid atherosclerotic plaques. In addition, high-resolution MRI of carotid plaques was performed at baseline, and at 12 and 24 months to test the effects of dalcetrapib on the evolution of plaque volume. Baseline FDG PET was used to select patients for study inclusion based on the magnitude of FDG uptake in atherosclerotic plaques. Only patients with an a priori-defined increased FDG uptake in plaques (TBR >1.6) were included in the trial [61, 62]. Patients with the highest reduction in FDG uptake after 6 months of treatment had the highest increase in circulating HDL under treatment and presented with a subsequent reduction in plaque volume measured with MRI after 24 months of treatment [62]. This suggests that a reduction in FDG uptake in atherosclerotic plaques represents an early imaging marker that precedes regression of plaque volume and is associated with the efficacy of these specific drugs.

Other similar therapeutic trials including substudies with FDG PET imaging of plaques have been performed in recent years. In one of these studies, the effect of a p38 mitogen-activated protein kinase (p38MAPK) inhibitor, BMS-582949, on atherosclerotic plaque inflammation in patients with documented atherosclerosis was evaluated [52]. Patients were randomly assigned to treatment with BMS-582949 (100 mg/day), atorvastatin (80 mg/day) or placebo. Treatment with BMS-582949 for 12 weeks did not reduce arterial inflammation or hs-CRP compared to placebo, whereas intensification of statin therapy significantly decreased arterial inflammation [52]. In another trial, the effects of VIA-2291, a potent inhibitor of arachidonate 5-lipoxygenase, a key enzyme in the synthesis of leukotrienes, on arterial inflammation was investigated in comparison with placebo [63]. VIA-2291 has been shown to reduce hs-CRP and noncalcified coronary plaque volume following an acute coronary syndrome. Arterial inflammation was again quantified using FDG PET and TBR. However, treatment with VIA-2291 was not associated with a significant difference in inflammation compared with placebo following 6 weeks and 24 weeks of treatment, or with a significant reduction in hs-CRP from baseline [63]. Finally, the effects of rilapladib (an inhibitor of lipoprotein-associated phospholipase A2, also known as platelet-activating factor acetylhydrolase) on arterial inflammation were investigated using FDG PET imaging in a multicentre, randomized, placebo-controlled study [64]. No significant reductions in FDG TBR were observed in the index vessel in the group treated with rilapladib [64]. Furthermore, lipoprotein-associated phospholipase A2 inhibition by rilapladib also did not enhance platelet aggregation [64, 65].

*Section summary and recommendations:*

- Measurement of FDG uptake in plaques is highly reproducible between PET studies. This translates into the possibility of detecting significant changes in FDG uptake with a relatively small group of patients.
- Initial FDG PET imaging can be used to screen patients entering a clinical study and select only those with increased FDG uptake in atherosclerotic plaques, who are at the highest risk of a cardiovascular event and might benefit the most from the tested drug.
- Changes in FDG uptake can be detected as early as 3 – 4 months after initiation of drug treatment, in contrast to changes in plaque volume, which occur later (12 – 24 months).
- Monitoring of the evolution of FDG uptake after initiation of a new treatment can give a hint as to the beneficial or deleterious effects of the drug on inflammatory activity in atherosclerotic plaques, even though FDG uptake in plaques is only a surrogate marker for the effects of drugs on plaque stabilization.
- Recommendations regarding the arterial territories to be analysed depending on the different study settings are given in Table 1.

## Perspectives in atherosclerotic plaque imaging

### PET/MRI for imaging of atherosclerotic plaques

Most current clinical PET systems are now combined with CT. These systems allow successive CT and PET acquisitions. CT acquisitions are used to generate tissue attenuation maps for the correction of the PET acquisition. In addition, CT images can be fused with the PET data to help localize areas accumulating radiotracer. CT images allow localization of the principal organs in a patient, but may be insufficient to identify precisely tissue structures in some clinical situations. These limitations have stimulated the development of combined PET/MRI systems. The main advantage of these systems is to associate the high tissue contrast of MRI with PET imaging [66]. Combined PET/MRI systems may offer several advantages for plaque imaging over PET/CT. First, imaging the arteries and/or the arterial plaque with CT requires angiography with injection of iodinated contrast agent, whereas with MRI this detection can be performed without paramagnetic contrast agents with dedicated sequences and "simply" using the intrinsic contrast of moving protons (i.e. blood). Second, as described previously, MRI allows arterial wall imaging with high contrast and spatial resolution. Hence, PET/MRI could offer the possibility to detect simultaneously both morphological and biological characteristics of atherosclerotic plaques. This will however require the implementation of MR acquisitions protocols and surface coils dedicated to vascular wall imaging in a combined PET/MRI system. Third, the evaluation of radiotracer uptake in coronary atherosclerotic plaques is difficult because of constant respiratory and cardiac motion during PET acquisition. The development of prospective respiratory and cardiac gating with MRI during PET acquisition could strongly improve the accuracy of PET measurements in coronary arteries. In summary, promising clinical applications are foreseen in the field of atherosclerotic plaque imaging for combined PET/MRI acquisition but such applications require the development of dedicated innovative imaging protocols in association with physicists [67].

### New PET tracers for atherosclerosis imaging

An important limitation of FDG PET studies is, however, the relatively nonspecific nature of FDG uptake. In particular, myocardial and skeletal muscle FDG uptake is frequent and often hampers precise analysis of coronary and carotid arteries. In addition, imaging with FDG requires patients to fast for at least 6 h before injection of the radiotracer to limit uptake by peripheral muscle and is often associated with poor image contrast in diabetic patients.

Therefore, new radiotracers more specifically targeting high-risk plaques might be interesting alternatives to FDG for the evaluation of atherosclerotic plaques with PET. In this context, several radiotracers such as ^11^C-choline or ^18^F-choline and ^68^Ga-DOTATATE, which bind to or accumulate in inflammatory cells, have been evaluated in patients for the detection of inflammation in atherosclerotic plaques [68–71]. The absence of any background signal in the myocardium facilitates the detection of radiotracer uptake in coronary arteries. For example, Rominger et al. were able to identify ^68^Ga-DOTATATE uptake in the left anterior descending artery in 70 patients imaged for oncological indications [71]. More recently, Folke Pederson et al. evaluated the intensity of ^64^Cu-DOTATATE uptake with PET/MRI in ten atherosclerotic plaques of patients scheduled for carotid endarterectomy [72]. Significantly higher ^64^Cu-DOTATATE uptake was detected in plaques ipsilateral to the ischaemic event than in the contralateral carotid artery [72].

^18^F-sodium fluoride (NaF) is a positron-emitting bone-seeking agent that reflects blood flow and remodelling of bone. Therefore, NaF has attracted interest for the imaging of calcified alterations in the arterial wall and in arterial plaques. In a feasibility study, Derlin et al. retrospectively evaluated imaging data obtained in 75 patients undergoing whole-body NaF PET/CT [73]. NaF uptake was observed at 254 sites in 57 patients (76 %) and calcification was observed at 1,930 sites in 63 patients (84 %) [73]. Colocalization of radiotracer accumulation and calcification was observed in 223 areas of uptake (88 %). Interestingly, only 12 % of all arterial calcification sites showed increased radiotracer uptake, indicating that NaF might be much more sensitive in detecting small calcified lesions, so-called spotty calcifications, and active calcium deposition than CT [73]. It is known that both active and passive mechanisms of calcium deposition may explain arterial calcification and that plaque calcification is an active process akin to bone formation [73]. Therefore, NaF uptake might be indicative of ongoing active mineral deposition in atherosclerotic lesions. Such plaques might not represent stable, non-progressive stages of disease [73]. Irkle et al. have been able to confirm this assumption as they showed a selective and specific high affinity of NaF for calcified deposits within arterial plaques [74]. Most intriguingly, NaF was able to distinguish between areas of macrocalcification and "active" microcalcification, the latter indicative of nascent calcification and active unstable atherosclerosis [74].

The same group later investigated the correlation between NaF accumulation in the common carotid arteries of neurologically asymptomatic patients with cardiovascular risk factors and carotid calcified plaque burden [75]. They included 269 oncological patients who underwent NaF PET/CT. NaF uptake in the common carotid arteries was observed at 141 sites in 94 patients (34.9 %) and showed colocalization with calcification in all atherosclerotic lesions. NaF uptake was significantly associated with age, male sex, hypertension and hypercholesterolaemia [75]. The presence of calcified plaque correlated significantly with these risk factors and also with diabetes, history of smoking, and prior cardiovascular events. Furthermore, there was also a highly significant correlation between the NaF uptake and the number of cardiovascular risk factors [75].

Joshi et al. reported the intriguing results of a prospective clinical trial of arterial NaF PET imaging [76]. Two groups of patients, 40 with myocardial infarction and 40 with stable angina, underwent both NaF and FDG PET/CT as well as invasive coronary angiography. NaF uptake was compared with histology of carotid endarterectomy specimens from patients with symptomatic carotid disease, and with intravascular ultrasonography in patients with stable angina [76]. In 37 patients (93 %) with myocardial infarction, the highest coronary NaF uptake was found in the culprit plaque. Interestingly, in contrast to the findings with NaF, which is not physiologically taken up by the myocardium, coronary FDG uptake was commonly obscured by myocardial uptake and where discernible there were no differences between the culprit and nonculprit plaques [76]. At the sites of all carotid plaque ruptures, significant NaF uptake was observed, which was associated with histological evidence of active calcification, macrophage infiltration, apoptosis and necrosis [76]. Plaques with focal NaF uptake were seen in 18 (45 %) of the patients with stable angina. Intravascular ultrasonography in these patients revealed that plaques with increased NaF uptake had more high-risk features, including positive remodelling, microcalcification and a necrotic core, than those without NaF uptake [76]. These promising results indicate that NaF PET imaging might be a sensitive method for identifying and localizing ruptured and high-risk coronary plaques.

## Summary/statement

In the past 10 years, several studies have shown that FDG PET imaging can reliably noninvasively evaluate the degree of inflammation present in atherosclerotic plaques. Patients with the highest FDG uptake in plaques show a higher rate of cardiovascular events and a worse prognosis than patients with low arterial FDG uptake. Hence, the FDG signal detected in plaques seems relevant to the evaluation of patients with atherosclerotic disease. One important limitation for the wider use of FDG PET in plaque imaging is the large variety of imaging protocols and quantification methods among centres. The aim of this position paper was to review the different parameters that can be chosen for atherosclerosis PET imaging and, based on the existing literature and the expertise of different European centres involved in plaque imaging, to define the "optimal" imaging protocol for plaque imaging with PET. Standardization of acquisition protocols for plaque imaging with PET would facilitate comparison of publications in the field and the setting up of meta-analyses and multicentre trials including patients from several imaging centres in Europe. An important challenge for the next years will be to evaluate whether the information extracted from PET imaging could play a role in the management of patients with atherosclerotic plaques. For this purpose, clear threshold or diagnostic criteria will need to be defined. Again, standardization of PET imaging protocols will be critical to ensure the reproducibility and robustness of the chosen values among different imaging centres.

In the future, algorithms for, among other things, respiratory gating and resolution recovery, but mainly the use of hybrid vascular PET/MRI, might provide a unique opportunity to combine morphological and functional evaluation of atherosclerotic plaques. In addition, precise delineation of plaque contours with high-resolution MRI might offer a more accurate correction of PVE and improved quantification of radiotracer accumulation in atherosclerotic plaques. Furthermore, FDG has the advantage of wide availability but has important limitations for plaque imaging including the need for fasting, interaction with blood glucose levels, and background signal in circulating blood and muscles. Development of new PET radiotracers more specific as markers of plaque vulnerability would greatly facilitate atherosclerosis imaging and stimulate the implementation of this technique.
